# Bis[μ-2,2′-dimethyl-1,1′-(oxydiethyl­ene)bis­(1*H*-benzimidazole)-κ^2^ *N* ^3^:*N* ^3′^]bis­[bis­(4-methoxy­benzoato-κ^2^ *O*,*O*′)cadmium(II)]

**DOI:** 10.1107/S1600536810009505

**Published:** 2010-03-20

**Authors:** Dian-Ying Zhao

**Affiliations:** a Weifang Vocational College, Weifang 261041, People’s Republic of China

## Abstract

The title complex, [Cd_2_(C_8_H_7_O_3_)_4_(C_20_H_22_N_4_O)_2_], forms a dimer of the paddle-wheel type, located on a crystallographic inversion centre. The Cd^II^ ion is hexa­coordinated by four carboxylate O atoms [Cd⋯O = 2.280 (2)–2.404 (2) Å] from two chelating 4-methoxy­benzoate anions, and two N atoms [Cd⋯N = 2.313 (2) and 2.332 (2) Å] from one chelating 2,2′-dimethyl-3,3′-(oxydiethyl­ene)bis­(1*H*-benzimidazole) ligand. In the crystal, mol­ecules are linked by a weak inter­molecular C—H⋯O hydrogen bond and an inter­molecular C—H⋯π inter­action.

## Related literature

For a related structure, see: Zhao *et al.* (2002 ▶). For bis­(imid­azole) ligands with –CH_2_– spacers as *N*-donor bridging ligands, see: Hoskins *et al.* (1997 ▶).
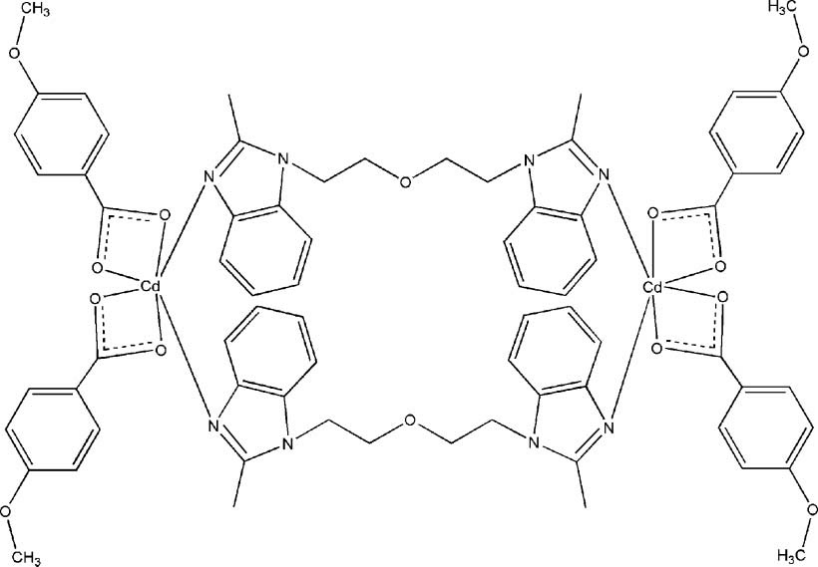


## Experimental

### 

#### Crystal data

[Cd_2_(C_8_H_7_O_3_)_4_(C_20_H_22_N_4_O)_2_]
*M* *_r_* = 1498.18
Triclinic, 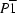

*a* = 9.0379 (5) Å
*b* = 13.8130 (8) Å
*c* = 13.9361 (6) Å
α = 88.143 (4)°
β = 86.539 (4)°
γ = 74.863 (4)°
*V* = 1676.11 (15) Å^3^
*Z* = 1
Mo *K*α radiation
μ = 0.71 mm^−1^
*T* = 293 K
0.28 × 0.24 × 0.21 mm

#### Data collection

Oxford Diffraction Gemini R Ultra diffractometer
Absorption correction: multi-scan (*CrysAlis CCD*; Oxford Diffraction, 2006 ▶) *T*_min_ = 0.831, *T*_max_ = 0.902
12519 measured reflections
7555 independent reflections
4356 reflections with *I* > 2σ(*I*)
*R*_int_ = 0.027

#### Refinement

*R*[*F* ^2^ > 2σ(*F* ^2^)] = 0.034
*wR*(*F* ^2^) = 0.051
*S* = 0.85
7555 reflections
433 parameters
H-atom parameters constrained
Δρ_max_ = 0.38 e Å^−3^
Δρ_min_ = −0.34 e Å^−3^

### 

Data collection: *CrysAlis CCD* (Oxford Diffraction, 2006 ▶); cell refinement: *CrysAlis CCD*; data reduction: *CrysAlis RED* (Oxford Diffraction, 2006 ▶); program(s) used to solve structure: *SHELXS97* (Sheldrick, 2008 ▶); program(s) used to refine structure: *SHELXL97* (Sheldrick, 2008 ▶); molecular graphics: *ORTEP-3* (Farrugia, 1997 ▶) and *DIAMOND* (Brandenburg, 1998 ▶); software used to prepare material for publication: *SHELXL97*.

## Supplementary Material

Crystal structure: contains datablocks I, global. DOI: 10.1107/S1600536810009505/lx2137sup1.cif

Structure factors: contains datablocks I. DOI: 10.1107/S1600536810009505/lx2137Isup2.hkl

Additional supplementary materials:  crystallographic information; 3D view; checkCIF report

## Figures and Tables

**Table 1:** Hydrogen-bond geometry (Å, °) *Cg* is the centroid of the C10–C15 benzene ring.

*D*—H⋯*A*	*D*—H	H⋯*A*	*D*⋯*A*	*D*—H⋯*A*
C21—H21⋯O7^i^	0.93	2.50	3.333 (3)	149
C6—H6⋯*Cg*^ii^	0.93	2.76	3.684 (5)	170

Symmetry codes: (i) 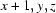; (ii) 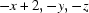.

**Fig. 1.**
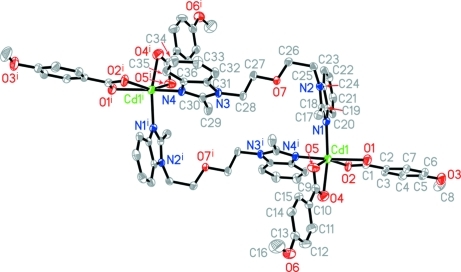
The molecular structure of the title compound with the atom numbering scheme. Displacement ellipsoids are drawn at the 30% probability level. H atoms have been omitted for clarity. [Symmetry code: (i) -x+1, -y+1, -z+1.]

**Fig. 2.**
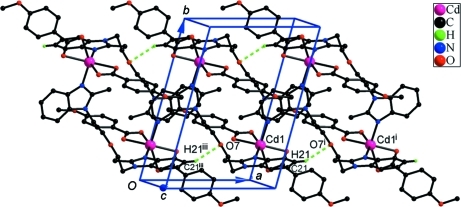
C–H···O interactions (dotted lines) in the title compound. [Symmetry code: (i) x+1, y, z; (iii) x-1, y, z.]

**Fig. 3.**
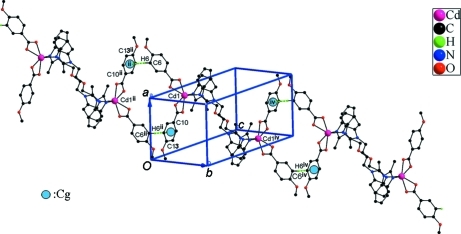
C–H···π interactions (dotted lines) in the title compound. [Symmetry code: (ii) -x+2, -y, -z; (iv) -x+1, -y+1, -z+1.]

## supplementary crystallographic information

### Comment

Bis(imidazole) ligands with –CH_2_- spacers are a good candidate for N-donor bridging ligand (Hoskins *et al.*, 1997). Up to now, 2,2'-bis(2-methyl-1H-benzimidazole)ether ligand, as a flexible ligand, is rarely investigated in constructing coordination polymers.

In the title compound (Fig. 1), the Cd^II^ ion is hexacoordinated by four O atoms from two chelating 4-methoxybenzoate anions, and two N atoms from one chelating 2,2'-bis(2-methyl-1H-benzimidazole)ether ligand. The Cd–O distances are found in the range from 2.280 (2) to 2.404 (2) Å, which is similar to previous report (Zhao *et al.*, 2002). The Cd–N distances are 2.313 (2) and 2.332 (2) Å, respectively. The crystal structure (Fig. 2) is stabilized by a weak intermolecular C–H···O hydrogen bond between the benzene H atom of 2-methyl-1H-benzimidazole ring and the O atom of diethyl ether group, with a C21–H21···O7 (Table 1 & Fig. 2). The crystal structure is further stabilized by an inetrmolecular C–H···π interaction between the aryl H atom of 4-methoxybenzoate group and the benzene ring of neighboring 4-methoxybenzoate group, with a C6-H6···Cg (Table 1 & Fig. 3; Cg is the centroid of the C10-C15 benzene ring).

### Experimental

An aqueous solution (10 ml) of 4-methoxybenzoic acid (0.072 g, 0.4 mmol ), 2,2'-bis(2-methylbenzimidazole)ether (0.065 g, 0.2 mmol) and Cd(Ac)_2_ (0.046 g, 0.2 mmol) was added in and sealed in 18 ml Teflon-lined stainless steel container. The container was heated to 140 °C and held at that temperature for 72 h, then cooled to room temperature at a rate of 10 °C/h. And then the title compound was isolated.

### Refinement

C-bound H-atoms were geometrically positioned (C–H 0.93 Å) and refined using a riding model, with U_iso_ = 1.2U_eq_ (C).

### Crystal data

**Table:**

[Cd_2_(C_8_H_7_O_3_)_4_(C_20_H_22_N_4_O)_2_]	*Z* = 1
*M**_r_* = 1498.18	*F*(000) = 768
Triclinic, *P*1	*D*_x_ = 1.484 Mg m^−^^3^
Hall symbol: -P 1	Mo *K*α radiation, λ = 0.71073 Å
*a* = 9.0379 (5) Å	Cell parameters from 4356 reflections
*b* = 13.8130 (8) Å	θ = 2.8–29.2°
*c* = 13.9361 (6) Å	µ = 0.71 mm^−^^1^
α = 88.143 (4)°	*T* = 293 K
β = 86.539 (4)°	Block, colorless
γ = 74.863 (4)°	0.28 × 0.24 × 0.21 mm
*V* = 1676.11 (15) Å^3^	

### Data collection

**Table:**

Oxford Diffraction Gemini R Ultra diffractometer	7555 independent reflections
Radiation source: fine-focus sealed tube	4356 reflections with *I* > 2σ(*I*)
graphite	*R*_int_ = 0.027
Detector resolution: 10.0 pixels mm^-1^	θ_max_ = 29.2°, θ_min_ = 2.8°
ω scan	*h* = −12→7
Absorption correction: multi-scan (*CrysAlis CCD*; Oxford Diffraction, 2006)	*k* = −18→16
*T*_min_ = 0.831, *T*_max_ = 0.902	*l* = −18→17
12519 measured reflections	

### Refinement

**Table:**

Refinement on *F*^2^	Primary atom site location: structure-invariant direct methods
Least-squares matrix: full	Secondary atom site location: difference Fourier map
*R*[*F*^2^ > 2σ(*F*^2^)] = 0.034	Hydrogen site location: inferred from neighbouring sites
*wR*(*F*^2^) = 0.051	H-atom parameters constrained
*S* = 0.85	*w* = 1/[σ^2^(*F*_o_^2^) + (0.0113*P*)^2^] where *P* = (*F*_o_^2^ + 2*F*_c_^2^)/3
7555 reflections	(Δ/σ)_max_ < 0.001
433 parameters	Δρ_max_ = 0.38 e Å^−^^3^
0 restraints	Δρ_min_ = −0.33 e Å^−^^3^

### Special details

**Table:**

Geometry. All esds (except the esd in the dihedral angle between two l.s. planes) are estimated using the full covariance matrix. The cell esds are taken into account individually in the estimation of esds in distances, angles and torsion angles; correlations between esds in cell parameters are only used when they are defined by crystal symmetry. An approximate (isotropic) treatment of cell esds is used for estimating esds involving l.s. planes.
Refinement. Refinement of *F*^2^ against ALL reflections. The weighted *R*-factor wR and goodness of fit *S* are based on *F*^2^, conventional *R*-factors *R* are based on *F*, with *F* set to zero for negative *F*^2^. The threshold expression of *F*^2^ > σ(*F*^2^) is used only for calculating *R*-factors(gt) etc. and is not relevant to the choice of reflections for refinement. *R*-factors based on *F*^2^ are statistically about twice as large as those based on *F*, and *R*- factors based on ALL data will be even larger.

### Fractional atomic coordinates and isotropic or equivalent isotropic displacement parameters (Å^2^)

**Table:**

	*x*	*y*	*z*	*U*_iso_*/*U*_eq_	
Cd1	0.94281 (2)	0.230266 (15)	0.254883 (14)	0.04683 (7)	
C1	1.2095 (3)	0.1039 (2)	0.19577 (17)	0.0473 (7)	
C2	1.3622 (3)	0.03887 (18)	0.16114 (16)	0.0422 (7)	
C3	1.4938 (3)	0.06938 (19)	0.16937 (16)	0.0490 (7)	
H3	1.4870	0.1311	0.1966	0.059*	
C4	1.6363 (3)	0.0106 (2)	0.13810 (17)	0.0583 (8)	
H4	1.7241	0.0326	0.1440	0.070*	
C5	1.6461 (3)	−0.0806 (2)	0.09832 (18)	0.0557 (8)	
C6	1.5159 (3)	−0.11242 (19)	0.08944 (17)	0.0561 (8)	
H6	1.5231	−0.1742	0.0624	0.067*	
C7	1.3742 (3)	−0.05289 (18)	0.12062 (16)	0.0505 (7)	
H7	1.2864	−0.0748	0.1142	0.061*	
C8	1.9160 (4)	−0.1138 (3)	0.0596 (2)	0.1036 (13)	
H8A	2.0000	−0.1668	0.0352	0.155*	
H8B	1.9367	−0.0965	0.1226	0.155*	
H8C	1.9039	−0.0562	0.0175	0.155*	
C9	0.7323 (4)	0.29694 (19)	0.1234 (2)	0.0550 (8)	
C10	0.6093 (3)	0.33063 (18)	0.05549 (19)	0.0491 (7)	
C11	0.6409 (4)	0.3618 (2)	−0.0378 (2)	0.0682 (9)	
H11	0.7408	0.3620	−0.0579	0.082*	
C12	0.5249 (5)	0.3923 (2)	−0.1003 (2)	0.0759 (10)	
H12	0.5475	0.4127	−0.1625	0.091*	
C13	0.3752 (4)	0.3931 (2)	−0.0725 (2)	0.0659 (9)	
C14	0.3418 (3)	0.3637 (2)	0.0201 (2)	0.0629 (9)	
H14	0.2415	0.3649	0.0406	0.075*	
C15	0.4595 (3)	0.33262 (18)	0.08161 (18)	0.0543 (8)	
H15	0.4364	0.3120	0.1437	0.065*	
C16	0.1153 (5)	0.4292 (3)	−0.1140 (3)	0.1410 (19)	
H16A	0.0536	0.4509	−0.1683	0.212*	
H16B	0.0815	0.4765	−0.0632	0.212*	
H16C	0.1053	0.3645	−0.0920	0.212*	
C17	0.6274 (3)	0.14017 (18)	0.39082 (17)	0.0476 (7)	
H17A	0.5531	0.1211	0.4344	0.071*	
H17B	0.6538	0.0940	0.3386	0.071*	
H17C	0.5849	0.2067	0.3660	0.071*	
C18	0.7663 (3)	0.13819 (16)	0.44216 (18)	0.0371 (6)	
C19	0.9933 (3)	0.14764 (15)	0.47397 (17)	0.0336 (6)	
C20	1.1423 (3)	0.15861 (16)	0.47225 (19)	0.0418 (7)	
H20	1.1887	0.1781	0.4162	0.050*	
C21	1.2181 (3)	0.13993 (18)	0.5554 (2)	0.0504 (7)	
H21	1.3179	0.1465	0.5555	0.060*	
C22	1.1499 (3)	0.11128 (18)	0.6402 (2)	0.0529 (7)	
H22	1.2042	0.1004	0.6958	0.064*	
C23	1.0033 (3)	0.09874 (17)	0.64291 (19)	0.0449 (7)	
H23	0.9583	0.0780	0.6989	0.054*	
C24	0.9266 (3)	0.11808 (15)	0.55947 (17)	0.0340 (6)	
C25	0.6691 (2)	0.08745 (16)	0.60518 (16)	0.0413 (6)	
H25A	0.7209	0.0370	0.6505	0.050*	
H25B	0.6005	0.0591	0.5709	0.050*	
C26	0.5759 (3)	0.17790 (17)	0.65981 (16)	0.0439 (7)	
H26A	0.5033	0.1582	0.7055	0.053*	
H26B	0.6430	0.2066	0.6950	0.053*	
C27	0.3805 (3)	0.32728 (17)	0.63788 (17)	0.0456 (7)	
H27A	0.4265	0.3686	0.6754	0.055*	
H27B	0.3131	0.2990	0.6804	0.055*	
C28	0.2916 (3)	0.38904 (16)	0.55925 (16)	0.0417 (7)	
H28A	0.3613	0.4138	0.5153	0.050*	
H28B	0.2443	0.3472	0.5233	0.050*	
C29	0.3390 (3)	0.59244 (19)	0.6021 (2)	0.0642 (9)	
H29A	0.4130	0.5382	0.5709	0.096*	
H29B	0.3777	0.6072	0.6610	0.096*	
H29C	0.3205	0.6507	0.5606	0.096*	
C30	0.1930 (3)	0.56328 (18)	0.62326 (17)	0.0418 (7)	
C31	0.0241 (3)	0.47285 (17)	0.62709 (16)	0.0356 (6)	
C32	−0.0560 (3)	0.40092 (18)	0.62173 (18)	0.0490 (7)	
H32	−0.0118	0.3390	0.5941	0.059*	
C33	−0.2046 (3)	0.4258 (2)	0.6594 (2)	0.0625 (8)	
H33	−0.2628	0.3796	0.6572	0.075*	
C34	−0.2700 (3)	0.5184 (2)	0.7009 (2)	0.0595 (8)	
H34	−0.3709	0.5322	0.7259	0.071*	
C35	−0.1908 (3)	0.58992 (19)	0.70604 (17)	0.0482 (7)	
H35	−0.2357	0.6517	0.7338	0.058*	
C36	−0.0400 (3)	0.56591 (16)	0.66778 (15)	0.0349 (6)	
N1	0.8906 (2)	0.16039 (13)	0.40196 (13)	0.0367 (5)	
N2	0.7832 (2)	0.11225 (13)	0.53661 (14)	0.0344 (5)	
N3	0.1729 (2)	0.47365 (13)	0.59854 (13)	0.0386 (5)	
N4	0.0680 (2)	0.62167 (13)	0.66601 (13)	0.0386 (5)	
O1	1.0920 (2)	0.07181 (13)	0.19366 (12)	0.0593 (5)	
O2	1.20357 (19)	0.18917 (13)	0.22786 (12)	0.0616 (5)	
O3	1.7801 (2)	−0.14625 (15)	0.06458 (14)	0.0859 (7)	
O4	0.8677 (2)	0.30016 (15)	0.10123 (13)	0.0741 (6)	
O5	0.6979 (2)	0.26483 (14)	0.20601 (13)	0.0668 (6)	
O6	0.2704 (3)	0.42284 (17)	−0.14127 (14)	0.0963 (8)	
O7	0.49629 (16)	0.24959 (11)	0.59312 (10)	0.0405 (4)	

### Atomic displacement parameters (Å^2^)

**Table:**

	*U*^11^	*U*^22^	*U*^33^	*U*^12^	*U*^13^	*U*^23^
Cd1	0.04789 (13)	0.04216 (11)	0.04792 (12)	−0.00858 (9)	0.00742 (9)	−0.00793 (9)
C1	0.056 (2)	0.0430 (17)	0.0361 (15)	−0.0029 (15)	0.0046 (14)	−0.0003 (13)
C2	0.0511 (18)	0.0391 (15)	0.0299 (14)	−0.0010 (13)	0.0037 (13)	−0.0019 (12)
C3	0.060 (2)	0.0428 (16)	0.0369 (15)	−0.0009 (15)	−0.0005 (14)	−0.0064 (13)
C4	0.0528 (19)	0.068 (2)	0.0498 (17)	−0.0067 (16)	−0.0032 (14)	−0.0045 (16)
C5	0.054 (2)	0.059 (2)	0.0409 (16)	0.0074 (17)	0.0022 (15)	0.0010 (15)
C6	0.075 (2)	0.0405 (16)	0.0442 (17)	0.0004 (16)	0.0034 (16)	−0.0085 (14)
C7	0.0609 (19)	0.0439 (16)	0.0419 (15)	−0.0066 (14)	0.0048 (14)	−0.0046 (13)
C8	0.055 (2)	0.148 (3)	0.091 (3)	0.003 (2)	0.010 (2)	−0.020 (2)
C9	0.068 (2)	0.0412 (17)	0.053 (2)	−0.0072 (15)	−0.0030 (18)	−0.0066 (15)
C10	0.068 (2)	0.0361 (15)	0.0391 (16)	−0.0071 (14)	−0.0003 (16)	−0.0015 (13)
C11	0.085 (3)	0.072 (2)	0.051 (2)	−0.0273 (19)	0.0034 (18)	−0.0011 (17)
C12	0.114 (3)	0.072 (2)	0.0429 (19)	−0.028 (2)	0.000 (2)	0.0098 (17)
C13	0.091 (3)	0.0497 (19)	0.0435 (19)	0.0071 (18)	−0.0110 (19)	−0.0019 (15)
C14	0.066 (2)	0.066 (2)	0.0431 (18)	0.0052 (16)	0.0005 (16)	−0.0039 (16)
C15	0.066 (2)	0.0508 (18)	0.0351 (15)	0.0040 (15)	0.0013 (16)	0.0025 (14)
C16	0.099 (4)	0.198 (5)	0.084 (3)	0.038 (3)	−0.027 (2)	0.016 (3)
C17	0.0413 (17)	0.0509 (16)	0.0511 (16)	−0.0130 (13)	−0.0025 (13)	−0.0019 (14)
C18	0.0364 (16)	0.0291 (14)	0.0437 (16)	−0.0040 (11)	−0.0011 (13)	−0.0089 (12)
C19	0.0294 (15)	0.0241 (13)	0.0464 (16)	−0.0047 (11)	−0.0006 (13)	−0.0077 (12)
C20	0.0369 (16)	0.0325 (14)	0.0556 (17)	−0.0090 (12)	0.0040 (14)	−0.0066 (13)
C21	0.0314 (16)	0.0420 (16)	0.078 (2)	−0.0070 (13)	−0.0111 (16)	−0.0064 (16)
C22	0.0484 (19)	0.0469 (17)	0.0625 (19)	−0.0069 (14)	−0.0207 (15)	0.0007 (15)
C23	0.0390 (17)	0.0416 (16)	0.0514 (17)	−0.0058 (13)	−0.0041 (14)	0.0026 (13)
C24	0.0308 (15)	0.0232 (13)	0.0455 (16)	−0.0013 (11)	−0.0040 (13)	−0.0037 (12)
C25	0.0354 (15)	0.0378 (14)	0.0472 (15)	−0.0054 (12)	0.0020 (12)	0.0068 (13)
C26	0.0364 (15)	0.0487 (16)	0.0426 (15)	−0.0049 (13)	0.0024 (12)	−0.0002 (14)
C27	0.0359 (15)	0.0441 (16)	0.0506 (16)	0.0005 (12)	0.0033 (13)	−0.0089 (14)
C28	0.0370 (15)	0.0353 (14)	0.0485 (15)	−0.0026 (12)	0.0063 (13)	−0.0086 (13)
C29	0.0430 (17)	0.0524 (17)	0.098 (2)	−0.0163 (14)	0.0126 (16)	−0.0176 (17)
C30	0.0304 (15)	0.0378 (15)	0.0560 (16)	−0.0082 (12)	0.0053 (13)	−0.0032 (13)
C31	0.0322 (14)	0.0345 (14)	0.0391 (14)	−0.0075 (12)	−0.0001 (11)	0.0008 (12)
C32	0.0457 (17)	0.0370 (15)	0.0637 (18)	−0.0106 (13)	0.0043 (14)	−0.0079 (14)
C33	0.0524 (19)	0.0510 (18)	0.091 (2)	−0.0266 (15)	0.0065 (17)	−0.0072 (17)
C34	0.0380 (17)	0.0600 (19)	0.080 (2)	−0.0164 (15)	0.0138 (15)	−0.0012 (17)
C35	0.0434 (17)	0.0414 (15)	0.0552 (17)	−0.0063 (13)	0.0122 (14)	−0.0034 (13)
C36	0.0353 (15)	0.0328 (14)	0.0341 (14)	−0.0054 (12)	0.0015 (12)	0.0015 (11)
N1	0.0355 (13)	0.0345 (11)	0.0408 (12)	−0.0106 (10)	0.0035 (11)	−0.0064 (10)
N2	0.0261 (12)	0.0313 (11)	0.0435 (13)	−0.0039 (9)	0.0001 (10)	−0.0002 (10)
N3	0.0336 (12)	0.0297 (11)	0.0479 (12)	−0.0015 (9)	0.0050 (10)	−0.0039 (10)
N4	0.0316 (12)	0.0336 (11)	0.0490 (12)	−0.0075 (10)	0.0091 (10)	−0.0069 (10)
O1	0.0546 (13)	0.0521 (12)	0.0682 (13)	−0.0111 (10)	0.0139 (11)	−0.0117 (10)
O2	0.0597 (13)	0.0456 (11)	0.0735 (13)	−0.0047 (9)	0.0152 (10)	−0.0232 (10)
O3	0.0643 (15)	0.0859 (15)	0.0865 (15)	0.0171 (13)	0.0079 (13)	−0.0158 (13)
O4	0.0643 (15)	0.0898 (16)	0.0635 (13)	−0.0134 (12)	0.0025 (11)	−0.0008 (12)
O5	0.0669 (14)	0.0808 (15)	0.0548 (12)	−0.0223 (11)	−0.0142 (10)	0.0164 (11)
O6	0.119 (2)	0.1003 (18)	0.0488 (13)	0.0132 (16)	−0.0250 (15)	0.0070 (13)
O7	0.0341 (10)	0.0372 (9)	0.0416 (9)	0.0048 (8)	0.0035 (8)	−0.0005 (8)

### Geometric parameters (Å, °)

**Table:**

Cd1—O5	2.280 (2)	C18—N1	1.328 (3)
Cd1—O2	2.286 (2)	C18—N2	1.360 (3)
Cd1—N1	2.313 (2)	C19—N1	1.384 (3)
Cd1—N4^i^	2.332 (2)	C19—C20	1.392 (3)
Cd1—O4	2.383 (2)	C19—C24	1.400 (3)
Cd1—O1	2.404 (2)	C20—C21	1.365 (3)
Cd1—C1	2.682 (2)	C20—H20	0.9300
Cd1—C9	2.687 (3)	C21—C22	1.394 (3)
C1—O1	1.255 (3)	C21—H21	0.9300
C1—O2	1.261 (3)	C22—C23	1.378 (3)
C1—C2	1.498 (3)	C22—H22	0.9300
C2—C3	1.373 (3)	C23—C24	1.373 (3)
C2—C7	1.380 (3)	C23—H23	0.9300
C3—C4	1.385 (3)	C24—N2	1.376 (3)
C3—H3	0.9300	C25—N2	1.462 (2)
C4—C5	1.373 (3)	C25—C26	1.512 (3)
C4—H4	0.9300	C25—H25A	0.9700
C5—C6	1.372 (4)	C25—H25B	0.9700
C5—O3	1.377 (3)	C26—O7	1.418 (3)
C6—C7	1.383 (3)	C26—H26A	0.9700
C6—H6	0.9300	C26—H26B	0.9700
C7—H7	0.9300	C27—O7	1.419 (2)
C8—O3	1.410 (4)	C27—C28	1.504 (3)
C8—H8A	0.9600	C27—H27A	0.9700
C8—H8B	0.9600	C27—H27B	0.9700
C8—H8C	0.9600	C28—N3	1.460 (2)
C9—O4	1.255 (3)	C28—H28A	0.9700
C9—O5	1.271 (3)	C28—H28B	0.9700
C9—C10	1.476 (4)	C29—C30	1.488 (3)
C10—C15	1.374 (3)	C29—H29A	0.9600
C10—C11	1.394 (3)	C29—H29B	0.9600
C11—C12	1.375 (4)	C29—H29C	0.9600
C11—H11	0.9300	C30—N4	1.325 (2)
C12—C13	1.382 (4)	C30—N3	1.355 (3)
C12—H12	0.9300	C31—C32	1.380 (3)
C13—O6	1.366 (4)	C31—N3	1.382 (3)
C13—C14	1.379 (4)	C31—C36	1.389 (3)
C14—C15	1.378 (4)	C32—C33	1.372 (3)
C14—H14	0.9300	C32—H32	0.9300
C15—H15	0.9300	C33—C34	1.389 (3)
C16—O6	1.411 (4)	C33—H33	0.9300
C16—H16A	0.9600	C34—C35	1.369 (3)
C16—H16B	0.9600	C34—H34	0.9300
C16—H16C	0.9600	C35—C36	1.392 (3)
C17—C18	1.476 (3)	C35—H35	0.9300
C17—H17A	0.9600	C36—N4	1.391 (3)
C17—H17B	0.9600	N4—Cd1^i^	2.3316 (18)
C17—H17C	0.9600		
			
O5—Cd1—O2	153.09 (6)	H17B—C17—H17C	109.5
O5—Cd1—N1	95.61 (7)	N1—C18—N2	111.6 (2)
O2—Cd1—N1	107.43 (7)	N1—C18—C17	124.4 (2)
O5—Cd1—N4^i^	100.00 (6)	N2—C18—C17	124.0 (2)
O2—Cd1—N4^i^	94.51 (6)	N1—C19—C20	130.8 (2)
N1—Cd1—N4^i^	88.28 (6)	N1—C19—C24	109.5 (2)
O5—Cd1—O4	55.99 (6)	C20—C19—C24	119.7 (2)
O2—Cd1—O4	99.60 (7)	C21—C20—C19	118.0 (2)
N1—Cd1—O4	151.45 (7)	C21—C20—H20	121.0
N4^i^—Cd1—O4	98.77 (7)	C19—C20—H20	121.0
O5—Cd1—O1	110.51 (7)	C20—C21—C22	121.7 (2)
O2—Cd1—O1	55.85 (6)	C20—C21—H21	119.1
N1—Cd1—O1	92.78 (6)	C22—C21—H21	119.1
N4^i^—Cd1—O1	149.19 (7)	C23—C22—C21	121.1 (3)
O4—Cd1—O1	94.88 (6)	C23—C22—H22	119.4
O5—Cd1—C1	134.16 (8)	C21—C22—H22	119.4
O2—Cd1—C1	27.98 (7)	C24—C23—C22	117.2 (2)
N1—Cd1—C1	102.11 (7)	C24—C23—H23	121.4
N4^i^—Cd1—C1	122.23 (8)	C22—C23—H23	121.4
O4—Cd1—C1	97.40 (7)	C23—C24—N2	132.5 (2)
O1—Cd1—C1	27.89 (7)	C23—C24—C19	122.3 (2)
O5—Cd1—C9	28.14 (7)	N2—C24—C19	105.2 (2)
O2—Cd1—C9	126.59 (8)	N2—C25—C26	112.38 (18)
N1—Cd1—C9	123.67 (8)	N2—C25—H25A	109.1
N4^i^—Cd1—C9	100.99 (7)	C26—C25—H25A	109.1
O4—Cd1—C9	27.85 (7)	N2—C25—H25B	109.1
O1—Cd1—C9	103.90 (7)	C26—C25—H25B	109.1
C1—Cd1—C9	117.52 (8)	H25A—C25—H25B	107.9
O1—C1—O2	121.8 (2)	O7—C26—C25	108.62 (18)
O1—C1—C2	119.6 (2)	O7—C26—H26A	110.0
O2—C1—C2	118.5 (3)	C25—C26—H26A	110.0
O1—C1—Cd1	63.63 (12)	O7—C26—H26B	110.0
O2—C1—Cd1	58.28 (12)	C25—C26—H26B	110.0
C2—C1—Cd1	176.4 (2)	H26A—C26—H26B	108.3
C3—C2—C7	118.5 (2)	O7—C27—C28	107.24 (18)
C3—C2—C1	120.6 (2)	O7—C27—H27A	110.3
C7—C2—C1	120.9 (3)	C28—C27—H27A	110.3
C2—C3—C4	121.7 (2)	O7—C27—H27B	110.3
C2—C3—H3	119.2	C28—C27—H27B	110.3
C4—C3—H3	119.2	H27A—C27—H27B	108.5
C5—C4—C3	119.0 (3)	N3—C28—C27	111.16 (18)
C5—C4—H4	120.5	N3—C28—H28A	109.4
C3—C4—H4	120.5	C27—C28—H28A	109.4
C6—C5—C4	120.2 (2)	N3—C28—H28B	109.4
C6—C5—O3	114.9 (3)	C27—C28—H28B	109.4
C4—C5—O3	124.9 (3)	H28A—C28—H28B	108.0
C5—C6—C7	120.3 (3)	C30—C29—H29A	109.5
C5—C6—H6	119.9	C30—C29—H29B	109.5
C7—C6—H6	119.9	H29A—C29—H29B	109.5
C2—C7—C6	120.4 (3)	C30—C29—H29C	109.5
C2—C7—H7	119.8	H29A—C29—H29C	109.5
C6—C7—H7	119.8	H29B—C29—H29C	109.5
O3—C8—H8A	109.5	N4—C30—N3	112.5 (2)
O3—C8—H8B	109.5	N4—C30—C29	124.4 (2)
H8A—C8—H8B	109.5	N3—C30—C29	123.00 (18)
O3—C8—H8C	109.5	C32—C31—N3	132.2 (2)
H8A—C8—H8C	109.5	C32—C31—C36	122.7 (2)
H8B—C8—H8C	109.5	N3—C31—C36	105.0 (2)
O4—C9—O5	120.3 (3)	C33—C32—C31	116.3 (2)
O4—C9—C10	121.5 (3)	C33—C32—H32	121.8
O5—C9—C10	118.3 (3)	C31—C32—H32	121.8
O4—C9—Cd1	62.46 (17)	C32—C33—C34	121.6 (2)
O5—C9—Cd1	57.83 (15)	C32—C33—H33	119.2
C10—C9—Cd1	176.0 (2)	C34—C33—H33	119.2
C15—C10—C11	117.4 (3)	C35—C34—C33	122.2 (2)
C15—C10—C9	121.5 (3)	C35—C34—H34	118.9
C11—C10—C9	121.1 (3)	C33—C34—H34	118.9
C12—C11—C10	120.2 (3)	C34—C35—C36	117.0 (2)
C12—C11—H11	119.9	C34—C35—H35	121.5
C10—C11—H11	119.9	C36—C35—H35	121.5
C11—C12—C13	121.2 (3)	C31—C36—N4	110.24 (18)
C11—C12—H12	119.4	C31—C36—C35	120.2 (2)
C13—C12—H12	119.4	N4—C36—C35	129.5 (2)
O6—C13—C14	124.9 (3)	C18—N1—C19	105.8 (2)
O6—C13—C12	115.9 (3)	C18—N1—Cd1	133.21 (19)
C14—C13—C12	119.2 (3)	C19—N1—Cd1	120.40 (15)
C15—C14—C13	118.9 (3)	C18—N2—C24	107.93 (19)
C15—C14—H14	120.5	C18—N2—C25	127.4 (2)
C13—C14—H14	120.5	C24—N2—C25	124.6 (2)
C10—C15—C14	123.0 (3)	C30—N3—C31	107.50 (17)
C10—C15—H15	118.5	C30—N3—C28	126.2 (2)
C14—C15—H15	118.5	C31—N3—C28	126.0 (2)
O6—C16—H16A	109.5	C30—N4—C36	104.68 (18)
O6—C16—H16B	109.5	C30—N4—Cd1^i^	125.24 (16)
H16A—C16—H16B	109.5	C36—N4—Cd1^i^	129.08 (12)
O6—C16—H16C	109.5	C1—O1—Cd1	88.48 (14)
H16A—C16—H16C	109.5	C1—O2—Cd1	93.74 (16)
H16B—C16—H16C	109.5	C5—O3—C8	118.3 (3)
C18—C17—H17A	109.5	C9—O4—Cd1	89.69 (18)
C18—C17—H17B	109.5	C9—O5—Cd1	94.03 (17)
H17A—C17—H17B	109.5	C13—O6—C16	117.5 (3)
C18—C17—H17C	109.5	C26—O7—C27	112.89 (16)
H17A—C17—H17C	109.5		
			
O5—Cd1—C1—O1	−37.75 (19)	C24—C19—N1—Cd1	171.44 (12)
O2—Cd1—C1—O1	177.0 (2)	O5—Cd1—N1—C18	5.74 (19)
N1—Cd1—C1—O1	72.59 (16)	O2—Cd1—N1—C18	−160.18 (18)
N4^i^—Cd1—C1—O1	168.27 (14)	N4^i^—Cd1—N1—C18	105.63 (19)
O4—Cd1—C1—O1	−86.45 (16)	O4—Cd1—N1—C18	0.3 (3)
C9—Cd1—C1—O1	−66.10 (17)	O1—Cd1—N1—C18	−105.19 (19)
O5—Cd1—C1—O2	145.23 (14)	C1—Cd1—N1—C18	−131.74 (19)
N1—Cd1—C1—O2	−104.42 (15)	C9—Cd1—N1—C18	3.6 (2)
N4^i^—Cd1—C1—O2	−8.75 (17)	O5—Cd1—N1—C19	−163.82 (15)
O4—Cd1—C1—O2	96.54 (15)	O2—Cd1—N1—C19	30.26 (16)
O1—Cd1—C1—O2	−177.0 (2)	N4^i^—Cd1—N1—C19	−63.93 (15)
C9—Cd1—C1—O2	116.88 (15)	O4—Cd1—N1—C19	−169.23 (14)
O2—C1—C2—C3	3.7 (4)	O1—Cd1—N1—C19	85.25 (15)
O1—C1—C2—C7	4.6 (4)	C1—Cd1—N1—C19	58.70 (16)
O2—C1—C2—C7	−176.5 (2)	C9—Cd1—N1—C19	−166.00 (14)
C7—C2—C3—C4	0.1 (4)	N1—C18—N2—C24	0.2 (2)
C1—C2—C3—C4	179.9 (2)	C17—C18—N2—C24	179.51 (19)
C2—C3—C4—C5	−0.3 (4)	N1—C18—N2—C25	177.09 (17)
C3—C4—C5—C6	0.3 (4)	C17—C18—N2—C25	−3.6 (3)
C3—C4—C5—O3	−179.9 (2)	C23—C24—N2—C18	−178.7 (2)
C4—C5—C6—C7	−0.1 (4)	C19—C24—N2—C18	−0.6 (2)
O3—C5—C6—C7	−179.9 (2)	C23—C24—N2—C25	4.2 (3)
C3—C2—C7—C6	0.2 (3)	C19—C24—N2—C25	−177.59 (17)
C1—C2—C7—C6	−179.6 (2)	C26—C25—N2—C18	−92.4 (3)
C5—C6—C7—C2	−0.2 (4)	C26—C25—N2—C24	84.0 (3)
O5—Cd1—C9—O4	178.7 (2)	N4—C30—N3—C31	0.0 (3)
O2—Cd1—C9—O4	−16.14 (18)	C29—C30—N3—C31	−178.7 (2)
N1—Cd1—C9—O4	−176.70 (13)	N4—C30—N3—C28	−173.5 (2)
N4^i^—Cd1—C9—O4	87.99 (15)	C29—C30—N3—C28	7.8 (4)
O1—Cd1—C9—O4	−73.70 (15)	C32—C31—N3—C30	−178.5 (3)
C1—Cd1—C9—O4	−47.55 (18)	C36—C31—N3—C30	0.8 (3)
O2—Cd1—C9—O5	165.18 (12)	C32—C31—N3—C28	−5.0 (4)
N1—Cd1—C9—O5	4.62 (17)	C36—C31—N3—C28	174.3 (2)
N4^i^—Cd1—C9—O5	−90.69 (14)	C27—C28—N3—C30	83.1 (3)
O4—Cd1—C9—O5	−178.7 (2)	C27—C28—N3—C31	−89.2 (3)
O1—Cd1—C9—O5	107.62 (14)	N3—C30—N4—C36	−0.7 (3)
C1—Cd1—C9—O5	133.77 (14)	C29—C30—N4—C36	177.9 (3)
O4—C9—C10—C15	−176.2 (2)	N3—C30—N4—Cd1^i^	168.72 (15)
O5—C9—C10—C15	3.4 (4)	C29—C30—N4—Cd1^i^	−12.6 (4)
O4—C9—C10—C11	3.8 (4)	C31—C36—N4—C30	1.2 (3)
O5—C9—C10—C11	−176.7 (2)	C35—C36—N4—C30	178.6 (3)
C15—C10—C11—C12	−0.5 (4)	C31—C36—N4—Cd1^i^	−167.67 (15)
C9—C10—C11—C12	179.5 (3)	C35—C36—N4—Cd1^i^	9.8 (4)
C10—C11—C12—C13	0.2 (5)	O2—C1—O1—Cd1	3.0 (3)
C11—C12—C13—O6	−178.7 (3)	C2—C1—O1—Cd1	−178.2 (2)
C11—C12—C13—C14	0.6 (4)	O5—Cd1—O1—C1	152.03 (15)
O6—C13—C14—C15	178.1 (3)	O2—Cd1—O1—C1	−1.69 (14)
C12—C13—C14—C15	−1.2 (4)	N1—Cd1—O1—C1	−110.92 (16)
C11—C10—C15—C14	0.0 (4)	N4^i^—Cd1—O1—C1	−19.6 (2)
C9—C10—C15—C14	179.9 (2)	O4—Cd1—O1—C1	96.60 (16)
C13—C14—C15—C10	0.9 (4)	C9—Cd1—O1—C1	123.35 (16)
N1—C19—C20—C21	−179.4 (2)	O1—C1—O2—Cd1	−3.1 (3)
C24—C19—C20—C21	−0.1 (3)	C2—C1—O2—Cd1	178.02 (19)
C19—C20—C21—C22	−0.4 (3)	O5—Cd1—O2—C1	−64.7 (2)
C20—C21—C22—C23	1.3 (4)	N1—Cd1—O2—C1	82.98 (15)
C21—C22—C23—C24	−1.6 (3)	N4^i^—Cd1—O2—C1	172.59 (14)
C22—C23—C24—N2	179.1 (2)	O4—Cd1—O2—C1	−87.72 (15)
C22—C23—C24—C19	1.1 (3)	O1—Cd1—O2—C1	1.69 (14)
N1—C19—C24—C23	179.2 (2)	C9—Cd1—O2—C1	−80.15 (16)
C20—C19—C24—C23	−0.3 (3)	C6—C5—O3—C8	171.9 (3)
N1—C19—C24—N2	0.8 (2)	C4—C5—O3—C8	−7.8 (4)
C20—C19—C24—N2	−178.71 (18)	O5—C9—O4—Cd1	1.3 (2)
N2—C25—C26—O7	60.9 (3)	C10—C9—O4—Cd1	−179.2 (2)
O7—C27—C28—N3	−177.81 (19)	O5—Cd1—O4—C9	−0.75 (14)
N3—C31—C32—C33	179.0 (2)	O2—Cd1—O4—C9	166.92 (14)
C36—C31—C32—C33	−0.2 (4)	N1—Cd1—O4—C9	5.8 (2)
C31—C32—C33—C34	−0.2 (4)	N4^i^—Cd1—O4—C9	−96.95 (15)
C32—C33—C34—C35	0.3 (5)	O1—Cd1—O4—C9	110.76 (15)
C33—C34—C35—C36	−0.1 (4)	C1—Cd1—O4—C9	138.70 (16)
C32—C31—C36—N4	178.2 (2)	O4—C9—O5—Cd1	−1.4 (2)
N3—C31—C36—N4	−1.2 (3)	C10—C9—O5—Cd1	179.12 (19)
C32—C31—C36—C35	0.5 (4)	O2—Cd1—O5—C9	−27.0 (2)
N3—C31—C36—C35	−178.9 (2)	N1—Cd1—O5—C9	−176.14 (14)
C34—C35—C36—C31	−0.3 (4)	N4^i^—Cd1—O5—C9	94.63 (14)
C34—C35—C36—N4	−177.5 (2)	O4—Cd1—O5—C9	0.74 (13)
N2—C18—N1—C19	0.3 (2)	O1—Cd1—O5—C9	−81.04 (15)
C17—C18—N1—C19	−179.0 (2)	C1—Cd1—O5—C9	−63.23 (18)
N2—C18—N1—Cd1	−170.34 (13)	C14—C13—O6—C16	3.7 (4)
C17—C18—N1—Cd1	10.3 (3)	C12—C13—O6—C16	−177.0 (3)
C20—C19—N1—C18	178.7 (2)	C25—C26—O7—C27	167.09 (19)
C24—C19—N1—C18	−0.7 (2)	C28—C27—O7—C26	−172.20 (19)
C20—C19—N1—Cd1	−9.2 (3)		

Symmetry codes: (i) −*x*+1, −*y*+1, −*z*+1.

### Hydrogen-bond geometry (Å, °)

**Table:**

Cg is the centroid of the C10–C15 benzene ring.

**Table:**

*D*—H···*A*	*D*—H	H···*A*	*D*···*A*	*D*—H···*A*
C21—H21···O7^ii^	0.93	2.50	3.333 (3)	149
C6—H6···Cg^iii^	0.93	2.76	3.684 (5)	170

Symmetry codes: (ii) *x*+1, *y*, *z*; (iii) −*x*+2, −*y*, −*z*.
